# Sex differences in clinical characteristics and outcomes after intracerebral haemorrhage: results from a 12-month prospective stroke registry in Nanjing, China

**DOI:** 10.1186/s12883-014-0172-5

**Published:** 2014-09-04

**Authors:** Junshan Zhou, Yingdong Zhang, Hisatomi Arima, Yanxia Zhao, Hongdong Zhao, Danni Zheng, Youyong Tian, Yukai Liu, Qing Huang, Jie Yang, for the Nanjing First Hospital stroke registry investigators

**Affiliations:** Department of Neurology, Nanjing First Hospital, Nanjing Medical University, 68 Changle Road, Nanjing, Jiangsu 210006 China; The George Institute for Global Health (H.A.), Royal Prince Alfred Hospital and University of Sydney, Sydney, NSW 2050 Australia

**Keywords:** Sex difference, Intracerebral haemorrhage, Outcome, Stroke registry

## Abstract

**Background:**

There is uncertainty surrounding the differences in outcomes after intracerebral haemorrhage (ICH) between men and women. This study aimed to investigate the sex differences in clinical characteristics, severity and outcomes of Chinese ICH patients.

**Methods:**

The Nanjing First Hospital stroke registry was a hospital-based registry of stroke patients with 1-year prospective follow-up. From 2004 to 2008, a total of 651 consecutively recruited patients with acute ICH were enrolled. Primary outcome was death or dependency defined as a modified Rankin Scale score of 3–6 at 12 months. Multivariable logistic regression analyses were performed to determine whether there were sex differences in clinical outcomes after ICH. Clinically important and biologically plausible risk factors of death or dependency were selected from available variables.

**Results:**

A total of 615 ICH patients were enrolled. There was no significant difference in age (63.5 ± 14.0 vs. 62.7 ± 12.7, p = 0.500) between women and men. At baseline, men were more likely to be current smokers (46.1% vs. 3.6%, P < 0.001) or current drinkers (35.4% vs. 3.6%, P < 0.001), but women had higher admission National Institute of Health Stroke Scale (NIHSS) scores than men (10 vs. 8, P = 0.039). Women also had higher rates of death or dependency at 3, 6, and 12 months (61.2% vs. 46.8%, P = 0.001; 56.7% vs. 45.3%, P = 0.009; and 51.8% vs. 44.1%, P = 0.065; respectively). After adjustment for age, existing hypertension and diabetes, prior stroke, previous ischemic heart disease, previous atrial fibrillation, current smoking and alcohol consumption status, pre-stroke dependency, onset-to-door time, admission NIHSS score, admission systolic blood pressure and location of bleeding, the association between the female gender and death or dependency remained statistical significant at 3 months [odds ratio (OR): 1.76; 95% confidence interval (CI): 1.07-2.89], but did not reach statistical significance at 6 months (OR: 1.59; 95% CI: 0.99-2.54) and 12 months (OR: 1.22; 95% CI: 0.77-1.95).

**Conclusions:**

In a Chinese population, women are more likely to be dead or dependent early after ICH than men. However, this gender difference gradually attenuates over the period of 12 months.

## Background

Stroke is one of the leading causes of mortality and adult disability worldwide [1]. In China, stroke has become the leading cause of mortality and adult disability [2]. Intracerebral haemorrhage (ICH) is the one of the most lethal types of stroke and is more frequently observed in Chinese than the Western populations [2].

In recent years, a growing interest in gender differences relating to stroke has been observed worldwide [3]. However, most studies on gender differences in stroke have focused on total or ischemic stroke [3–5], and few studies have separately explored gender differences in ICH, particularly concerning differences in functional outcomes [6–8]. In addition, the results from these studies have been conflicting. Some studies reported higher mortality in women after ICH [9–11], while other studies suggested the opposite [12,13] or no gender differences in post-ICH mortality [8,14,15]. These discrepancies might be attributable to different choices of study setting (population-based or hospital-based), study population (Asian or Western), inclusion criteria, analytical methods used and duration of follow-up. Furthermore, most of the previous studies were retrospective [9–13,15], and the follow-up period of the only prospective study was very short (less than 1 month) [14].

In order to assess the effect of gender differences in clinical characteristics, stroke severity and outcomes after ICH, we employed a hospital-based, large-scale, prospective, 12-month follow-up study of ICH patients in Nanjing, China.

## Methods

### Subjects

The Nanjing First Hospital stroke registry (NFHSR) was a hospital-based registry of stroke patients with 1-year prospective follow-up. Nanjing First Hospital is the only tertiary hospital in the Qinhuai District in Nanjing City (the Southeast urban region of China), there are 700,000 citizens in this district, and more than 80% stroke patients in this area are admitted to our hospital. All consecutive patients, admitted to the neurology department with acute stroke (ischemic stroke, intracerebral haemorrhage or subarachnoid haemorrhage) between August 2004 and August 2008, were invited to participate. In Nanjing First Hospital, more than 95% ICH patients are admitted to Neurology wards, and less than 5% ICH patients with larger hematoma volume and requirement of surgery were admitted to Neurosurgery ward. Patients were prospectively registered and followed up for 12 months after verbal or written informed consent were gained from each participant or their legal surrogate. Clinical diagnosis of stroke was made according to the WHO criteria and was confirmed by brain CT or MRI scans [16]. The present analyses included patients with acute ICH within 14 days of symptom onset who had a complete 12-month follow-up. In addition, primary intraventricular haemorrhage was excluded in these analyses, but intraventricular haemorrhage secondary to ICH was included. NFHSR was approved by the Nanjing First Hospital Ethics Committee for studies involving human subjects.

### Data collection

Detailed information on patient demographics (age and gender), risk factors, pre-stroke dependency, onset-to-door time (ODT), stroke severity, laboratory tests, and brain imaging data were collected. Risk factors included smoking and alcohol consumption status, presence of hypertension, diabetes and hypercholesterolemia, history of stroke, ischemic heart disease (including myocardial infarction and angina pectoris) and atrial fibrillation. Patients who had smoked >1 cigarette/day for more than one year were defined as current smokers. Patients who had consumed alcohol >50 ml/day for at least one year were classified as current drinkers. Hypertension was defined as systolic blood pressure (SBP) >140 mm Hg and/or diastolic blood pressure (DBP) >90 mm Hg or current use of antihypertensive agents. Diabetes was defined as fasting serum glucose level >7.0 mmol/L or current use of antidiabetic drugs. Hypercholesterolemia was defined as fasting serum cholesterol level >5.72 mmol/L or current use of lipid-lowering medicines. Pre-stroke dependency was defined as a modified Rankin Scale (mRS) score of 3–5 [17]. Severity of stroke was evaluated using the Glasgow Coma Scale (GCS) [18] and the National Institutes of Health Stroke Scale (NIHSS) [19]. Location of bleeding was defined as deep cerebral (periventricular white matter, caudate, globus pallidus, putamen, internal capsule, and thalamus), lobar, cerebellar and brain stem [20].

### Outcomes

Participants were followed up at 3, 6 and 12 months after ICH by telephone interview or questionnaire. Death was defined as the cumulative all-cause death. Dependency was defined as a mRS score of 3–5 [17].

The primary outcomes were death or dependency at 3, 6 and 12 months after ICH. The secondary outcomes were each component of the primary outcome (death and dependency separately) at 3, 6 and 12 months.

### Statistical analysis

Gender differences in baseline clinical characteristics and outcomes at 3, 6 and 12 months after ICH were evaluated using the Chi-square test for categorical variables and the Student *t*-test or Wilcoxon test for continuous variables. The gender differences in outcomes were ascertained by logistic regression models. Covariates including age, hypertension, diabetes, prior stroke, previous ischemic heart disease, previous atrial fibrillation, current smokers, current drinkers, pre-stroke dependency, ODT, admission NIHSS, admission SBP and location of bleeding were included in multivariable regression analyses. All statistical analyses were performed using SPSS version 16.0 (SPSS Inc., Chicago, IL) and a standard level of significance (p < 0.05) was used.

## Results

### Gender specific baseline clinical characteristics

**Figure 1:**
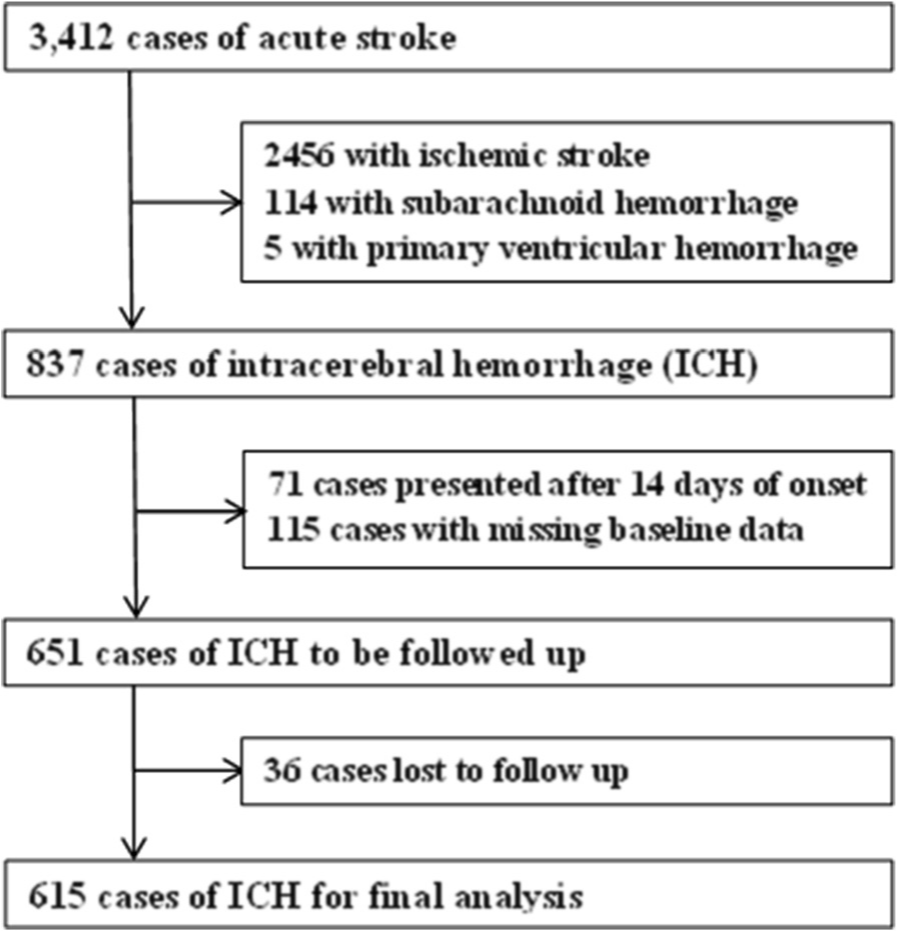
**Flow chart of patients’ selection.**

Figure 1 shows the flow chart of patients’ selection. Over the 5-year study period, 3412 consecutive participants with acute stroke were included in the NFHSR. After exclusion of patients who presented after 14 days of symptom onset and those with missing baseline data, 651 ICH patients were followed up for 12 months. Thirty six (5.5%) cases were lost to follow up, and the remaining 615 (94.5%) who had a complete 12 months follow-up were enrolled for the present analysis.

Table 1 shows the baseline clinical characteristics of the 615 ICH patients (220 [35.8%] women and 395 [64.2%] men). There was no significant difference in age (63.5 ± 14.0 vs. 62.7 ± 12.7, p = 0.500) between women and men. However, compared to the male group, the female group had a lower proportion of current smokers (3.6% vs. 46.1%, p < 0.001) and current drinkers (3.6% vs. 35.4%, p < 0.001). In addition, women had higher NIHSS scores (10 [4-17] vs. 8 [3-15], p = 0.039) than men. There were no statistical differences in pre-stroke dependency (11.8% vs. 9.1%, p = 0.286), ODT (6 [3-24] vs. 7 [3–48], p = 0.508), location of bleeding (P = 0.566) and other baseline clinical characteristics between women and men.

**Table 1:** **Gender specific baseline characteristics of intracerebral haemorrhage patients**

**Characteristics**	**Women (n = 220)**	**Men (n = 395)**	**P value**
**Demographics**			
Age (years)	63.5 ± 14.0	62.7 ± 12.7	0.500
**Medical history**			
Hypertension	120 (54.5)	215 (55.4)	0.830
Diabetes mellitus	16 (7.3)	27 (6.8)	0.838
Prior stroke	25 (11.4)	32 (8.1)	0.181
Ischemic heart disease	6 (2.7)	12 (3.0)	0.827
Atrial fibrillation	8 (3.6)	7 (1.8)	0.151
Current smoker	8 (3.6)	183 (46.1)	<0.001
Current drinker	8 (3.6)	140 (35.4)	<0.001
**Clinical features**			
Pre-stroke dependency^#^	26 (11.8)	36 (9.1)	0.286
Median OTD time (hours)	6 (3–24)	7 (3–48)	0.508
ODT ≤3 hours	73 (33.2)	131 (33.2)	0.997
SBP at admission (mmHg)	162.7 ± 31.2	162.7 ± 30.7	0.994
DBP at admission (mmHg)	94.3 ± 16.9	96.6 ± 17.8	0.118
Median NIHSS score†	10 (4–17)	8 (3–15)	0.039
NIHSS score >14	69 (31.4)	103 (26.1)	0.161
Median GCS score*	14 (11–15)	15 (11–15)	0.192
GCS score <9	45 (20.5)	74 (18.7)	0.605
Location of bleeding			
Basal ganglia	166 (75.5)	295 (74.7)	0.566
Lobar	38 (17.3)	80 (20.3)	
Cerebellum	5 (2.3)	5 (1.3)	
Brain stem	11 (5.0)	15 (3.8)	

Data are n (%), mean (SD), or median (IQR).

ODT, onset to door time; SBP, Systolic blood pressure; DBP, Diastolic blood pressure; NIHSS, National Institutes of Health Stroke Scale; GCS, Glasgow Coma Scale.

#Pre-stroke dependency defined as a score of 3–5 of the modified Rankin Scale.

†NIHSS scores can range from 0 (healthy) to 42 (coma with quadriplegia).*GCS scores can range from 3 (deep coma) to 15 (healthy).

### Gender specific outcomes at 3, 6, and 12 months after ICH

The outcomes at 3, 6, and 12 months after ICH are shown separately for women and men in Table 2.

**Table 2:** **Effects of gender (women vs. men) on outcomes at 3, 6, and 12 months after intracerebral haemorrhage**

**Outcomes**	**No of Outcomes (%)**		**Unadjusted OR**	**Adjusted OR 1** ^*^	**Adjusted OR 2** ^*^
	**Women**	**Men**	**(95%CI)**	**(95%CI)**	**(95%CI)**
**Death or dependency**					
3 month	123 (61.2)	174 (46.8)	1.79 (1.27-2.54)	1.85 (1.22-2.81)	1.76 (1.07-2.89)
6 month	119 (56.7)	169 (45.3)	1.58 (1.12-2.22)	1.64 (1.09-2.47)	1.59 (0.99-2.54)
12 month	114 (51.8)	174 (44.1)	1.37 (0.98-1.90)	1.34 (0.90-2.00)	1.22 (0.77-1.95)
**Death**					
3 month	57 (25.9)	90 (22.8)	0.88 (0.58-1.24)	0.90 (0.57-1.41)	1.16 (0.68-1.99)
6 month	61 (27.7)	100 (25.3)	0.88 (0.61-1.28)	0.92 (0.59-1.43)	1.18 (0.70-1.97)
12 month	64 (29.1)	115 (29.1)	1.00 (0.70-1.44)	1.05 (0.68-1.61)	1.38 (0.83-2.28)
**Dependency**					
3 month	65 (45.5)	84 (29.8)	2.00 (1.32-3.02)	2.19 (1.31-3.65)	1.94 (1.11-3.42)
6 month	58 (38.9)	69 (25.3)	1.88 (1.23-2.89)	2.17 (1.28-3.66)	2.02 (1.16-3.53)
12 month	50 (32.1)	59 (21.1)	1.77 (1.14-2.75)	1.76 (1.03-3.01)	1.58 (0.89-2.80)

OR, odds ratio; CI, confidence interval.

Dependency was defined as the modified Rankin Scale scores of 3–5.

*In multivariable analysis 1, odds ratios were adjusted for age, hypertension, diabetes, prior stroke, previous ischemic heart disease, previous atrial fibrillation, current smokers, current drinkers, pre-stroke dependency, onset-to-door time, admission systolic blood pressure and location of bleeding. *In multivariable analysis 2, odds ratios were adjusted for variables in multivariable analysis 1 and admission National Institutes of Health Stroke Scale (NIHSS) score.

**Figure 2:**
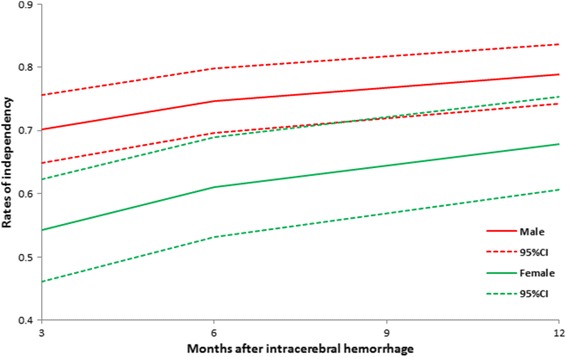
**Rates of independency after intracerebral haemorrhage by sex.**

Compared with men, women had higher rates of death or dependency at 3, 6 and 12 months after ICH (61.2% vs. 46.8%, P = 0.001; 56.7% vs. 45.3%, P = 0.009; and 51.8% vs. 44.1%, P = 0.065; respectively), but the gender differences in death or dependency attenuated gradually over the period of 12 months (Table 2 and Figure 2). After adjustment for potential confounding factors such as age, existing hypertension and diabetes, prior stroke, previous ischemic heart disease, previous atrial fibrillation, current smoking and alcohol consumption status, pre-stroke dependency, ODT, admission SBP and location of bleeding, the sex difference in death or dependency remained significant at 3 months [odds ratio (or): 1.85; 95% confidence interval (CI): 1.22-2.81] and 6 months (OR: 1.64; 95% CI: 1.09-2.47), but did not reach statistical significance at 12 months (OR: 1.34; 95% CI: 0.90-2.00) (Table 2). However, when additionally adjusted for the admission NIHSS, these associations remained significant only at 3 months (OR: 1.76; 95% CI: 1.07-2.89).

There were no clear differences in mortality between women and men (Table 2). In contrast, dependency was more frequently observed in women than in men at 3, 6 and 12 months after ICH (45.5% vs. 29.8%, 38.9% vs. 25.3% and 32.1% vs. 21.1%, respectively). These differences in dependency remained significant after adjustment for the same potential confounders such as age, existing hypertension and diabetes, prior stroke, previous ischemic heart disease, previous atrial fibrillation, current smoking and alcohol consumption status, pre-stroke dependency, ODT, admission SBP and location of bleeding at 3 months (OR:2.19; 95% CI:1.31-3.65), at 6 months (OR:2.17; 95% CI:1.28-3.66), and at 12 months (OR:1.76; 95% CI:1.03-3.01). However, when additionally adjusted for admission NIHSS, these associations remained significant only at 3 months (OR: 1.94; 95% CI: 1.11-3.42) and 6 months (OR: 2.02; 95% CI: 1.16-3.53).

## Discussion

We hypothesized that there would be differences in clinical characteristics and outcomes between female and male patients with ICH in China. Compared to men, women had higher admission NIHSS scores and were less likely to be current smokers or drinkers. Furthermore, women were more likely to be dead or dependent at 3 months after ICH. However, the gender difference in death or dependency gradually attenuated over the period of 12 months.

### Clinical characteristics

In our study, the female group had a lower proportion of current smokers and current drinkers than the male group, which is consistent with previous reports on the Chinese general population [21] and other hospital-based ICH study [11]. However, in contrast to prior investigations, our study demonstrated that women had more serious forms of ICH [11,14]. These discrepancies between the results of studies may be attributable to difference in ethnicity, study setting and inclusion criteria.

### Gender specific outcomes at 3, 6, and 12 months after ICH

#### Death or dependency

A few studies have explored gender differences in death or dependency after ICH [11,14,22,23], but there has been no data on the Chinese population. Our study suggested that women had an increased risk of death or dependency at 3 month after ICH, which may be driven by differences in dependency between the gender groups. The present analyses are consistent with the report from a hospital-based ICH registry in the United States [11]. But, some other hospital-based ICH registries in Turkey and in United States showed that there was no significant sex difference in death or dependency at discharge [14,23]. However, these hospital-based ICH registries’ sample size was not larger enough (320 patients), with short follow-up duration (less than 1 month), and were not adjusted for admission NIHSS score [14,23].

#### Death

Our data suggested that there was no significant sex difference in death at 3, 6 and 12 months after ICH, which is consistent with that of some previous prospective studies [8,14,15]. However, there were also other studies that showed a higher risk of death in women,[9–11] and studies that indicated a lower risk of death in women [12,13]. Unfortunately, many of these studies with different results were retrospective and did not adjust for other potential confounders.

#### Dependency

No previous study has separately explored the sex difference in dependency after ICH. Our analysis indicated that women had a higher risk of dependency at 3 and 6 months after ICH. The worse outcome in female is likely to be in part attributable to more severe ICH at baseline. In addition, community-based rehabilitation system in China is underdeveloped. The majority of rehabilitation services are provided by hospital located rehabilitation agencies and also costly. Due to employment and financial reasons, elderly Chinese women have less capability than men to afford Basic Health Insurance and consequently have less chance of gaining rehabilitation services [2,24]. It is warranted that the Chinese government should make a greater contribution towards the subsidy of women’s health care insurance, community-based rehabilitation systems and foster the development of social support to reduce stroke related declines in quality of life in Chinese women.

### Strengths and limitations

NFHSR has a number of strengths including the prospective design, 12-month follow-up, high follow-up rate (94.5%) and relatively large sample size (615 ICH patients). These strengths enabled us to explore the sex differences in outcomes among Chinese patients with ICH, which were not well-explored previously.

Some limitations should also be noted. First, NFHSR is a single-centre, urban setting, and teaching hospital-based stroke registry, not all ICH patients in the study area and in the hospital were covered. Furthermore, some patients with missing data or lost to follow up were excluded from the analysis. Therefore, there may be some selection bias in our registry and our findings may not be generalizable to the general ICH patients in China, especially those in rural areas. Second, there was no information on ICH hematoma volume in this registry. So we did not know if the hematoma volume had also influenced our results. Finally, there was a paucity of data on the management and care of ICH patients, which may have influenced the gender differences in ICH outcomes.

## Conclusions

In the Chinese population, women are more likely to be dead or dependent early after ICH than men, which may be driven by differences independency between gender groups. However, the gender differences in death or dependency gradually attenuated over a period of 12 months.

## Abbreviations

WHO: World Health Organization
NFHSR: Nanjing first hospital stroke registry
ICH: Intracerebral haemorrhage
NIHSS: National Institute of Health Stroke Scale
GCS: Glasgow coma scale
mRS: Modified rankin scale
ODT: Onset-to-door time
SBP: Systolic blood pressure
DBP: Diastolic blood pressure
CT: Computed tomography
MRI: Magnetic resonance imaging
OR: Odds ratio
CI: Confidence interval
